# Effects of antenatal care service utilization on maternal near miss in Gamo Gofa zone, southern Ethiopia: retrospective cohort study

**DOI:** 10.1186/s12884-021-03683-y

**Published:** 2021-03-16

**Authors:** Tayue Tateke Kebede, Wanzahun Godana, Mesfin Mamo Utaile, Yemisirach Berhanu Sebsibe

**Affiliations:** 1 School of Public Health and Community Medicine, Institute of Medicine, Sahlgrenska Academy, University of Gothenburg, Gothenburg, Sweden; 2 Department of Public Health, College of Medicine and Health Science, Arba Minch University, Arba Minch, Ethiopia; 3 Department of Nursing, College of Medicine and Health Science, Arba Minch University, Arba Minch, Ethiopia

**Keywords:** Maternal near miss, Antenatal care, Maternal health

## Abstract

**Background:**

Antenatal care (ANC) provides an opportunity to prevent, identify and intervene maternal health problems. Maternal near miss (MNM), as an indicator of maternal health, is increasingly gaining global attention to measure these problems. However, little has been done to measure the effect of ANC on MNM in Ethiopia. Therefore, this study is aimed at determining the effect of ANC on MNM and its associated predictors at Gamo Gofa zone, southern Ethiopia.

**Methods:**

Employing a retrospective cohort study design, 3 years data of 1440 pregnant mothers (480 ANC attendant and 960 non-attendant) were collected from all hospitals in the zone. Taking ANC visit as an exposure variable; we used a pretested checklist to extract relevant information from the study participants’ medical records. Characteristics of study participants, their ANC attendance status, MNM rates and associated predictors were determined.

**Results:**

Twenty-five (5.2%) ANC attendant and seventy-one (7.4%) non-attendant mothers experienced MNM, (X^2^ = 2,46, df = 2, *p* = 0.12). The incidence rates were 59.6 (95% CI: 40.6–88.2) and 86.1 (95%CI: 67.3–107.2)/1000 person-years for the ANC attendant and non-attendant mothers, respectively. Mothers who were living in rural areas had higher hazard ratio of experiencing MNM than those who were living in urban areas, with an adjusted hazard ratio (AHR) of 1.68 (95% CI, 1.01, 2.78).

**Conclusion:**

ANC attendance tended to reduce MNM. However, late initiation and loss to follow-up were higher in the current study. Therefore, on time initiation and consistent utilization of ANC are required.

## Background

Maternal near miss is an important indicator of maternal health status. Though maternal mortality has so far been considered as the main critical measure of maternal health status, recent research initiatives tend to focus on maternal morbidity. Thus, MNM is attracting more attention, especially when sample size is not large enough to calculate mortality ratio [1–3]. MNM is defined as an acute obstetric complication that immediately threatens a woman’s survival but do not result in her death either by chance or because of hospital care she receives during pregnancy, labor or within 6 weeks after termination of pregnancy or delivery [4].

It is more prevalent than maternal mortality in all countries though it is disproportionately higher in developing countries [5–7]. Globally, about 20 million MNM occurs every year, with the incidence range of less than 1 to more than 83 per 1000 live births [8–12]. Countries with high maternal mortality have large burden of MNM [13, 14]. For instance, in Liberia it was six times more common than maternal mortality [15].

Maternal health problem is a serious public health concern in Ethiopia [16, 17]. The country was one of the six countries that contributed about 50% of the maternal deaths worldwide in 2008 [18]. Three consecutive demographic and health surveys (EDHS 2000, 2005 and 2011) showed little reduction in maternal mortality ratio i.e. 871, 673 and 676 per 100,000, respectively. In the recent EDHS, maternal death represents 30% of all deaths of women aged 15–49. It is higher than the previous EDHSs’ figures [16, 19, 20]. Furthermore, the causes and factors of maternal mortality and morbidity are diverse and multifaceted [17, 21, 22]. Low utilization of the available maternal health interventions, including ANC services might constitute a vital part of the problem.

ANC attendance varies in different parts of the country [23–26]. The national ANC utilization coverage was 41% with the lowest rate in the Somali region (19.1%) and highest in Addis Ababa (94.2%) [27]. From the same survey, southern nations, nationalities and people’s region’s (SNNPR), where Gamo Gofa is located, ANC coverage was 39%.

As part of routine ANC services at hospitals, mothers get ultrasound examination, blood pressure checkup, blood group and Rh test, urine test, screening for diseases such as anemia and sexually transmitted infections (syphilis and HIV), iron tablets, deworming drugs, tetanus toxoid immunization and information on the sings of complications. Thus, ANC provides an opportunity to prevent, identify and intervene problems, and promote health of the mothers and their babies during pregnancy, delivery and at postpartum periods [28]. In the previous studies, lack of ANC visit was associated with poor pregnancy outcomes [29, 30]. Moreover, MNM was higher among mothers who had low ANC attendance [2, 31].

In Ethiopia, it is common to see many pregnant mothers seeking care after developing complications during pregnancy and/or childbirth, and often reach at health facilities in moribund conditions. However, little has been researched to measure the effects of ANC services to reduce MNM. Determining the effect of the service in preventing MNM and identifying associated predictors will help policy makers and health care providers to make evidence-based decisions to improve the service. Thus, this study is aimed at determining the effect of ANC visit on MNM and its associated predictors in Gamo Gofa zone, southern Ethiopia. The study hypothesized that ANC attendance reduces the risk of MNM.

## Methods

### Study design and settings

A retrospective cohort study design was employed. Three years data, from February 2011 to January 2014, were collected from all hospitals in Gamo Gofa zone. The zone has one general hospital and two district hospitals for the total population of 1,597,767. Since the hospitals started using the health management information system (HMIS) in December 2010, we could manage to have 3 years data.

The exposure variable was ANC visit. Mothers who had at least one ANC visit and received maternal health services during their recent pregnancy, childbirth or within 42 days of termination of the pregnancy at the hospitals within 3 years of the above stated period were the ANC attendant group. In comparison, those who had no ANC visit but received other maternal health services during their recent pregnancy, childbirth or within 42 days of termination of their pregnancy in the stated period at the hospitals were the non-ANC attendant group. Thus, considering the average gestational period of 280 days, the maximum follow-up period was 322 days. The study participants were assigned to each cohort considering their ANC follow-up history (ANC card) and medical records.

### Sample size and sampling procedure

All mothers who came to the hospitals to seek health care for their recent pregnancy during the study period were the source population. The sample size was determined using epi info version 7 considering 95% confidence level, 80% power and one to two ratios of ANC attendant and non-attendant groups. The variable taken to determine the sample size was MNM rate at a hospital in Bolivia, in which the rate for mothers with at least one ANC visit was 23.7% and for those with no ANC visit, it was 30.7% [2]. These rates were taken from a study conducted in another developing country that has relatively similar situation because we did not find similar studies in Ethiopia. Thus, the total sample size was 1440. For mothers with no ANC visit, the sample size was 960 while for those with at least one ANC visit, it was 480. The number of participants at each hospital was determined based on the proportion of mothers who visited the hospitals within the follow-up period.

Using the maternal and child health department registries, mothers were sorted out to ANC attendant and non-attendant groups. From each group, the registration number of the participant mothers were selected by simple random sampling technique (random number using SPSS 20). Subsequently, the selected mothers’ logbooks were identified and extracted from the card rooms.

### Data collection instruments and procedures

Data were collected through reviewing maternal care logbooks at the hospitals between February and June 2014. The review was done based on the checklist that has been developed by adopting from previous similar studies [4, 32, 33]. To ensure its validity and comprehensiveness, the checklist was pretested at Gidole hospital, which is found in an adjacent zone, before the actual data collection.

Data collectors were nurses and midwives who were working at maternal and child health (MCH) department of the hospitals. They received a training and supervised closely to ensure the quality of the data.

### Sample demographics

In this retrospective cohort study, 1440 pregnant mothers (480 ANC attendant and 960 non-attendant) were followed for 1255.7 person-years with respective mean of 319.3 ± 12.6 and 318.1 ± 16.4 for ANC attendant and non-attendant mothers. Their mean age was 25.5 ± 5.1 for ANC attendant and 25.4 ± 5.4 for non-attendant groups. The two groups were quite homogenous in their marital status (x^2^ = 0.35, df = 2, *p* = 0.838). However, their pregnancy (x^2^ = 38.5, df = 2, *P* < 0.001) and parity (x^2^ = 37.6, df = 2, *P* < 0.001) were not similar. For the ANC attendant group, ANC attendance was higher among mothers who resided in urban areas (x^2^ = 188.5, df = 2, *p* < 0.001) (Table 1).

**Table 1:** Background information of study participants at hospitals of Gamo Gofa zone, 2014

Variables	ANC attendant^a^	Non-ANC attendant^b^	Total	*P*- value
	Frequency (%)	Frequency (%)		
Residence				
Rural	138 (29.6)	633 (68.3)	771 (55.3)	
Urban	329 (70.4)	294 (31.7)	623 (44.7)	< 0.001
Marital status				
Married	459 (98.5)	920 (98.1)	1379 (98.2)	
Single	6 (1.3)	16 (1.7)	22 (1.6)	
Divorced	1 (0.2)	2 (0.2)	3 (0.2)	0.838
Pregnancy				
1	174 (36.6)	451 (48.4)	625 (44.5)	
2–4	262 (55.2)	354 (38.0)	616 (43.8)	< 0.001
> =5	39 (8.2)	126 (13.5)	165 (11.7)	
Parity				
0	–	24 (2.6)	24 (1.7)	
1	210 (44.3)	476 (51.0)	686 (48.8)	
2–4	231 (48.7)	324 (34.7)	555 (39.4)	< 0.001
> =5	33 (7.0)	109 (11.7)	142 (10.1)	

^a^480, ^b^960

### Case selection criteria

The disease-specific criteria used in previous studies [5] were employed for selecting near miss cases. It includes: (I) haemorrhage leading to shock; emergency hysterectomy; coagulation defects and/or blood transfusion of ≥2 l; (II) hypertensive disorders in pregnancy, including both eclampsia and severe preeclampsia with clinical/laboratory indications for termination of pregnancy to save the woman’s life; (III) dystocia; uterine rupture and impending rupture, e.g., prolonged obstructed labor with previous caesarean section; (IV) infection with hyperthermia or hypothermia or a clear source of infection; and clinical signs of septic shock and (V) severe anaemia (haemoglobin level < 7 g/dl). Mothers who experienced one of these problems under each category of the diseases were classified as near miss cases.

### Data analysis

Data were cleaned, edited, coded and entered to Epi Info version 7 before being exported to SPSS version 20 for further analysis. Descriptive statistics were calculated to determine frequencies and means of the study participants’ sociodemographic characteristics. Relative risks and incidence per 1000 person-years of MNM among the mothers who had ANC follow-up and those who hadn’t were determined. MNM incidence ratio was also computed to compare our finding with previous studies, since most of previous studies reported so. In addition, cox proportional hazard regression analysis was done to determine the effect of ANC visit and other associated predictors of MNM.

## Results

### History of ANC follow-up

**Fig. 1:**
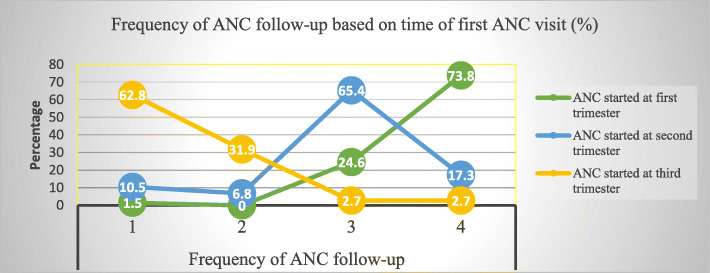
Distribution of ANC follow-up frequency based on the time at which the follow-up was started among ANC attendant mothers at hospitals of Gamo Gofa zone, 2014

Among the ANC attendant group, 140 (29.2%) mothers had four or more ANC visits. Meanwhile, those who had three, two and one ANC visits were 190 (39.6%), 52 (10.8%) and 98 (20.4), respectively. On the other hand, 130 (27.1%) mothers started their ANC visit at their 1st trimester. While the rest, 237(49.4%) and 113 (23.5%) of them started at their 2nd and 3rd trimester, respectively. Majority of the mothers (73.8%) who initiated ANC follow-up during their 1st trimester had at least four ANC visits. On the contrary, 72 (62.8%) of the mothers who started ANC follow-up during their 3rd trimester had only one ANC visit. In addition, loss-of follow-up was less prevalent among mothers who initiated ANC visits during their 1st trimester compared to those who started ANC visits during their 2nd and 3rd trimesters, (Fig. 1).

### Maternal near miss

In the current study, 25(5.2%) mothers in the ANC attendant and 71(7.4%) in the non-attendant groups experienced MNM (X^2^ = 2,46, df = 2, *p* = 0.12). The incidence rates found to be 59.6 (95% CI: 40.6–88.2) and 86.1 (95%CI: 67.3–107.2) per 1000 person-years for ANC attendant and non-attendant mothers, respectively. The corresponding incidence ratios were 53.0 and 86.2 per 1000 live births. Likewise, the relative risk among the ANC attendant group was 0.67 (95%CI, 0.43–1.04).

Hypertensive disorder and haemorrhage were the leading causes of MNM, (Table 2). The number of mothers with at least two MNM events were 2(8%) and 1(1.4%) among ANC-attendant and non-attendant mothers, respectively.

**Table 2:** Causes of maternal near miss among ANC attendant and non-attendant groups of mothers at hospitals of Gamo Gofa zone, 2014

Causes	ANC attendant	Non-ANC attendant
	Freq. (%)	Freq. (%)
Haemorrhage	5 (20)	23 (32)
Hypertensive disorder	13 (52)	21 (30)
Dystocia	3 (12)	13 (18)
Infection	3 (12)	10 (14)
Severe anaemia	1 (4)	4 (6)
Total	25 (100)	71 (100)

In the ANC attendant group, the maximum rate (14 out of the total 25) of near miss events was recorded among those who had three ANC visits. All of them started their visit at their 2nd trimester. On the other hand, among those mothers who had only one ANC visit and experienced MNM, 4 (80%) came to the hospitals after they developed pregnancy related complication.

Regardless of the ANC attendance status, the event of MNM was high among those who tended to deliver at home. Among those who experienced MNM, 12(48%) of mothers from the ANC attendant group came to the hospitals with pregnancy related complication, while the corresponding figure for non-attendant group was 22(31%). Thus, for the ANC attendant group, the relative risk of experiencing MNM when coming to the hospitals with pregnancy related complications was 20.17 (95%CI: 10.51–38.70). While for the non-attendant group, the relative risk of experiencing MNM when coming to the hospitals with this condition was 6.02 (95%CI: 3.85–9.41). On the other hand, six mothers who experienced MNM from non-attendant group came to the hospitals with complication of unsafe abortion.

### Survival status of the mothers

Regarding to status of the mothers after delivery, 6(0.4%) mothers were unstable (1 from ANC attendant and 5 from non-attendant groups), 6(0.4%) mothers died (1 from ANC attendant and 5 from non-attendant groups), 1426 (99.0%) mothers stable and the status of 2 (0.1%) mothers (both from non-attendant groups) was not documented. The MNM mortality ratio was 24:1 and 14:1 for ANC attendant and non-attendant mothers, respectively. Likewise, mortality index was 4.0 and 6.5% for ANC attendant and non-attendant mothers, respectively.

**Fig. 2:**
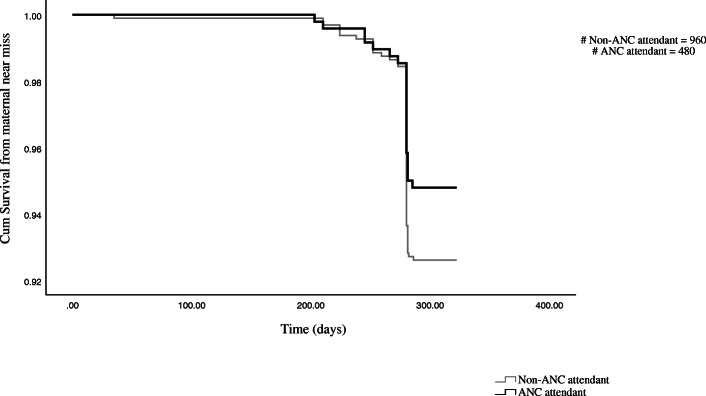
Survival curve of maternal near miss among ANC attendant and non-attendant mothers at hospitals of Gamo Gofa zone, 2014

The survival probability was slightly higher for the ANC attendant mothers as shown in Fig. 2 though it was not statistically significant, *p* = 0.12. For both groups, the survival probability was low around 40 weeks of pregnancy.

### Predictors of maternal near miss

For the ANC attendant group, parity was statistically significant predictor of MNM in the bivariate model (Table 3). Mothers who had one parity had lower hazard of experiencing MNM than those who had five, 0.23 (95% CI, 0.08, 0.71).

**Table 3:** Bivariate predictors of maternal near miss among ANC attendant and non-attendant groups of mothers at hospitals of Gamo Gofa zone, 2014

Variables	ANC attendant			Non-ANC attendant		
	Freq. (*N* = 480)	Hazard ratio (95% CI)	P	Freq. (*N* = 960)	Hazard ratio (95%CI)	P
Age		1.04 (0.97, 1.12)	0.255		1.06 (1.03, 1.10)	0.001
Residence						
Urban*	329	1		294	1	
Rural	138	1.34 (0.59, 3.04)	0.479	633	1.97 (1.08, 3.60)	0.028
Pregnancy						
1	174	0.34 (0.11, 1.03)	0.056	451	0.37 (0.19, 0.69)	0.002
2–4	262	0.34 (0.12, 0.95)	0.040	354	0.56 (0.32, 1.05)	0.072
≥ 5*	39	1		126	1	
Parity						
0	0	_		24	0.87 (0.25, 2.97)	0.817
1	210	0.23 (0.08, 0.71)	0.010	476	0.36 (0.20, 0.68)	0.001
2–4	231	0.32 (0.11, 0.90)	0.032	324	0.49 (0.26, 0.93)	0.028
≥ 5*	33	1		109	1	
History of chronic illness						
Yes	18	–		12	5.73 (2.09, 15.72)	0.001
No*	459	1		941	1	

*- Reference category

For the non-ANC attendant group, age, residence, number of pregnancies, parities and history of chronic illness were statistically significant predictors of MNM. Mothers who were living in rural areas were two times more likely to experience MNM than who were living in urban areas with the crude hazard ratio (CHR) of 1.97 (95% CI, 1.08–3.60). On the other hand, mothers who had 1 and 2–4 parities were less likely experienced MNM than those who had five or above parities with the respective CHR of 0.36 (95% CI, 0.20–0.68) and 0.49 (95% CI, 0.26–0.92).

In the multivariate cox regression analysis, ANC follow-up tended to be protective from MNM though it was not statistically significant, 0.86 (95%CI, 0.51, 1.45) (Table 4). Mothers who were living in rural area had higher adjusted hazard ratio (AHR) of experiencing MNM, 1.68 (95% CI, 1.01, 2.78) than those who were living in urban areas.

**Table 4:** multivariate predictors of maternal near miss at hospitals of Gamo Gofa zone, 2014

Variables	Freq. (*N* = 1440)	Hazard ratio (95% CI)	*P*
**Age**	1440	1.03 (0.99, 1.07)	0.144
**Residence**			
Urban*	609	1	
Rural	742	1.68 (1.01, 2.78)	0.045
**Order of Current pregnancy**			
1	600	1.49 (0.16, 13.88)	0.724
2–4	592	1.78 (0.24, 13.05)	0.572
≥ 5*	159	1	
**Parity**			
0	23	0.85 (0.07, 10.68)	0.901
1	656	0.33 (0.04, 3.09)	0.333
2–4	535	0.35 (0.05, 2.66)	0.312
≥ 5*	137	1	
**ANC follow-up**			
Yes	458	0.86 (0.51, 1.45)	0.572
No*	893	1	
**History of chronic illness**			
Yes	30	2.57 (0.92, 7.18)	0.071
No*	1321	1	

*- Reference category

## Discussion

In the current study, the incidence of MNM tended to be lower among mothers who had ANC visit. It was 59.6 (95% CI: 40.6–88.2) per 1000 person-years compared with 86.1 (95%CI: 67.3–107.2) per 1000 person-years of those who had no ANC visit. Likewise, the survival probability and relative risk of MNM were tended to be better for them.

It is worthy to note that this study has some limitations. Since we collected data from medical records, some outcomes could be underestimated and the variables that could possibly affect MNM might be overlooked due to unrecorded data. Besides, the current study considered only mothers who had contact with public hospitals. The information of mothers who utilized private health services were unobserved. Underestimation might have happened also because women with ANC visit could have better possibility of coming to the hospitals and had more near miss diagnosis than women without ANC visit.

One of the challenges in MNM measurement is lack of standardized approach [7, 34–37]. Finding uniform case identification criteria remained to be crucial task in the field, despite commendable efforts from World health organization (WHO) and other experts [38–40]. The WHO’s MNM criteria, which is one of the most accepted criteria in the high-income settings, is less feasible to apply and it underestimates the problem in the low-income settings [36, 37, 40, 41]. Other criteria have also their own drawbacks and choosing a criterion involves dealing with inevitable trade-offs. So, in the current study, considering the quality of the available data and context of the study setting, we opted to be conservative and took the disease specific criteria proposed by Filippi et al. [5, 42].

Mothers who had ANC visit tended to have better outcomes than their counterparts. The MNM mortality ratio of 24:1 versus 14:1 and mortality index of 4 versus 6.5 for mothers who had ANC visit and who had not, respectively, were indicators of positive effect of the intervention. In addition, though it is with marginal statistical significance, the relative risk of 0.67 and the better survival probability of the mothers are the signals that the ANC visit had a beneficial effect. The reason for the marginality of the effects of the routine ANC visit might include: A) the higher rate of loss to follow-up of ANC services and tendency to deliver at home. For instance, among mothers who had only one ANC visit and experienced MNM, 4 (80%) of them came to hospitals after they developed pregnancy related complications. This was in line with previous findings of higher rates of MNM for such cases [43]. B) Late initiation of ANC visit and C) low quality of the ANC service in the study setting [44, 45].

Late initiation of ANC visit and lost to follow-up were high among the mothers who had ANC follow-up in the current study. Only about a fourth (27%) of mothers started their ANC visit at their first trimester. It is quite consistent with previous findings in Ethiopia, but lower than reports from other developing countries [46–50]. Likewise, only 29% of the mothers who started ANC follow-up had four and above ANC visit. This finding is consistent with a recent national report and other findings from Ethiopia but much lower than reported elsewhere [27, 51–53]. Whatsoever the case, more than 70% of mothers in the ANC attendant group had inconsistent and low utilization of ANC services in the current study. Multifaceted and complexly arrayed factors might play role(s) for this to happen. Family income, experiences with previous utilization of ANC, mother’s education level, understanding about ANC service among mothers and their partners, perceived poor quality of the service and interactions with health care providers were among the major factors mentioned in previous studies [46, 47, 52, 54, 55]. In addition, the residence area of mothers influenced their ANC attendance in line with previous reports [53, 55, 56].

The current incidence of MNM is higher than previously reported findings from other countries. Even among the mothers who had ANC follow-up, the incidence ratio, 53.0 per 1000 live births, is higher than reported findings from Rwanda, Brazil, Bolivia and Iran [43, 57–59]. However, it is lower than a finding in a Brazilian study and much lower than finding from a study done in Debre Markos, Ethiopia [60, 61]. But, incidence rate among mothers who had no ANC visit was higher than the finding in the Brazilian study. Part of these differences could be attributed to methodological difference. On top of that, since the country’s maternal health problem is among the worst in the world, part of the difference with the first cases might be explained by the existing problem at the ground.

Aside from the influence of ANC attendance, the probability of experiencing MNM was predicted by other factors. Mothers who were living in rural areas had higher hazard probability of experiencing MNM than those who were living in urban areas. A previous study reported similar finding despite the difference of the statistical model [60]. Likewise, mothers who had no ANC attendance and had history of chronic illness had about six times more likely hazard probability of experiencing MNM than those who had not, as shown in the bivariate model. This finding is higher than previously reported findings [60]. In the bivariate cox regression model, mothers who had less than five parities and pregnancies had lower probability of experiencing MNM than those who had five and more in the ANC attendant and non-attendant groups. In line with this finding, mother who had higher parity were more likely experienced MNM than those who had low parity in the previous studies [60, 62].

In the current study, despite the inconsistent utilization and late initiation of the service, ANC attendance tended to reduce MNM. In the ANC attendant group, a somewhat higher hazard ratio of MNM was observed among mothers who started their follow-up at their second trimester or after and who did not use health institutional delivery. This indicates that on top of ensuring availability of the service, checking the utilization and the effect of the routine ANC service is essential to address maternal health problems.

## Conclusion

ANC attendance tended to reduce MNM. However, late initiation and loss to follow-up were higher among the ANC attendant groups in the current study. Therefore, interventions that promote on time initiation and consistent utilization of the services are required to optimize its effectiveness. In addition, further studies with prospective design are recommended to discern out more on the effect of the service on MNM.

## Data Availability

The datasets used and/or analysed during the current study are available from the corresponding author on reasonable request.

## Abbreviations

ANC: Antenatal care
AHR: Adjusted hazard ratio
CHR: Crude hazard ratio
EDHS: Ethiopian demographic and health surveys
SNNPR: Southern nations, nationalities and people’s region
Rh: Rhesus factor
HIV: Human immunodeficiency virus
HMIS: Health management information system
MCH: Maternal and child health
MNM: Maternal near miss
SPSS: Statistical Package for the Social Sciences
WHO: World Health Organization
